# Low Rate of Acquired Linezolid Resistance in Multidrug-Resistant Tuberculosis Treated With Bedaquiline-Linezolid Combination

**DOI:** 10.3389/fmicb.2021.655653

**Published:** 2021-05-03

**Authors:** Jian Du, Jingtao Gao, Yanhong Yu, Qingfeng Li, Guanghong Bai, Wei Shu, Mengqiu Gao, Yuhong Liu, Lu Wang, Yufeng Wang, Zhongtan Xue, Fengmin Huo, Liang Li, Yu Pang

**Affiliations:** ^1^Clinical Center on TB, Beijing Chest Hospital, Capital Medical University, Beijing Tuberculosis and Thoracic Tumor Research Institute, Beijing, China; ^2^Tuberculosis Laboratory, Shenyang Tenth People’s Hospital (Shenyang Chest Hospital), Shenyang, China; ^3^Department of Laboratory, Public Health and Clinical Center of Chengdu, Chengdu, China; ^4^Department of Laboratory, Shaanxi Provincial Tuberculosis Institute, Xi’an, China; ^5^Department of Tuberculosis, Beijing Chest Hospital, Capital Medical University, Beijing Tuberculosis and Thoracic Tumor Research Institute, Beijing, China; ^6^Beijing Key Laboratory on Drug-Resistant Tuberculosis Research, Department of Bacteriology and Immunology, Beijing Chest Hospital, Capital Medical University, Beijing Tuberculosis and Thoracic Tumor Research Institute, Beijing, China; ^7^Department of Laboratory Quality Control, Innovation Alliance on Tuberculosis Diagnosis and Treatment (Beijing), Beijing, China

**Keywords:** tuberculosis, linezolid, bedaquiline, susceptibility, multidrug-resistant

## Abstract

In this retrospective study in China, we aimed to: (1) determine the prevalence of linezolid (LZD) resistance among multidrug-resistant tuberculosis (MDR-TB)-infected patients; (2) monitor for dynamic LZD susceptibility changes during anti-TB treatment; and (3) explore molecular mechanisms conferring LZD resistance. A total of 277 MDR-TB patients receiving bedaquiline (BDQ)-containing regimens in 13 TB specialized hospitals across China were enrolled in the study. LZD and BDQ susceptibility rates were determined using the minimum inhibitory concentration (MIC) method, then DNA sequences of patient isolates were analyzed using Sanger sequencing to detect mutations conferring LZD resistance. Of 277 patients in our cohort, 115 (115/277, 41.5%) with prior LZD exposure yielded 19 (19/277, 6.9%) isolates exhibiting LZD resistance. The LZD resistance rate of LZD-exposed group isolates significantly exceeded the corresponding rate for non-exposed group isolates (*P* = 0.047). Genetic mutations were observed in 10 (52.6%, 10/19) LZD-resistant isolates, of which a Cys154Arg (36.8%, 7/19) substitution within ribosomal protein L3 was most prevalent. Analysis of sequential positive cultures obtained from 81 LZD-treated patients indicated that cultured organisms obtained from most patients (85.2%, 69/81) retained original LZD MIC values; however, organisms cultured later from two patients exhibited significantly increased MIC values that were attributed to the *rplC* substitution T460C. Overall, LZD resistance was detected in 6.9% of patients of an MDR-TB cohort in China. Low rate of acquired LZD resistance was noted in MDR-TB treated with BDQ-LZD combination.

## Introduction

Multidrug-resistant tuberculosis (MDR-TB), defined as disease caused by *Mycobacterium tuberculosis* complex (MTBC) strains with rifampicin and isoniazid resistance, poses a major challenge to TB elimination worldwide (Dheda et al., 2017; World Health Organization, 2019). The World Health Organization estimates that globally there were 484,000 MDR-TB cases in 2018, with the large majority of cases occurring in low- and middle-income countries, especially India, China, and Russia (World Health Organization, 2019). Due to initial drug resistance of MDR-TB bacilli to potent first-line anti-TB drugs, treatment of multidrug-resistant TB requires prolonged and complex administration of a limited number of second-line regimens that nevertheless are associated with poorer treatment outcomes (Bastos et al., 2017; Dheda et al., 2017). Alarmingly high rates of clinical failure have raised interest in newly developed and repurposed drugs that may improve treatment outcomes for MDR-TB patients (Tiberi et al., 2018).

Linezolid (LZD), a member of the oxazolidinone family of antibiotics, has been shown to improve MDR-TB patient outcomes in clinical trials (Schecter et al., 2010; Agyeman and Ofori-Asenso, 2016; Bolhuis et al., 2018). Based on clinical trial results, in 2018 WHO endorsed the use of LZD as a preferred agent for administration to all patients with MDR-TB (World Health Organization, 2018). Of note, results of a recent trial conducted in South Africa that evaluated a combination of linezolid, bedaquiline (BDQ), and pretomanid demonstrated favorable treatment outcomes in a high percentage of patients with highly drug-resistant forms of TB (Conradie et al., 2020). Taken together, the accumulating body of clinical data demonstrating LZD treatment benefits warrants an increasingly greater role for LZD as a cornerstone anti-TB agent for combating drug-resistant TB in national TB treatment programs (Sharma et al., 2017; Sharma et al., 2020).

Before LZD is used on a global scale, understanding mechanisms of LZD resistance is essential in order to guide formulation of optimal drug combinations with LZD. So far, mutations in ribosomal genes *23S rRNA* (encoding ribosomal RNA, *rrl*) and *rplC* (encoding the L3 protein) have been identified as the predominant molecular mechanisms associated with LZD resistance in MTB (Beckert et al., 2012; Zhang et al., 2014, 2016; Pi et al., 2019). Nevertheless, due to limited use of this costly drug, MTB resistance to LZD is still a rare phenomenon in clinical settings, with most LZD-resistant mutants identified only after *in vitro* selection (Zimenkov et al., 2017). Therefore, it would be meaningful to clarify potential pathways contributing to *in vivo* emergence of LZD resistance in patients after antibiotic exposure. With this goal in mind, we conducted a retrospective study to determine the prevalence of LZD resistance among MDR-TB patients in China, monitored dynamic LZD susceptibility changes during anti-TB treatment, and explored molecular mechanism(s) conferring LZD resistance in our cohort.

## Materials and Methods

### Study Design

A retrospective study was conducted in 13 TB specialized hospitals across China of MDR-TB patients receiving BDQ-containing regimens, of whom 91% had prior LZD treatment histories. Sputum specimens were collected from patients at baseline, 2, 4, 8, 12, 16, 20, and 24 weeks after treatment initiation, as previously reported (Gao et al., 2020). MTB isolates collected at baseline and at final follow-up for each patient were recovered by culturing specimens on Löwenstein-Jensen medium for 4 weeks at 37°C prior to *in vitro* drug susceptibility testing. Patient demographic and clinical characteristics were obtained from a database accessible via a user-friendly online portal^1^.

### *In vitro* Drug Susceptibility Testing

*In vitro* drug susceptibility of MTB isolates to LZD was determined using a Middlebrook 7H9 broth microdilution method and Thermo Fisher frozen microtiter plates. Briefly, 4-week-old fresh colonies were scraped from Löwenstein-Jensen (L-J) slants then cells were disrupted and suspended by vigorous mixing with glass beads. Each suspension of MTB cells was adjusted to a density equal to that of a 1.0 McFarland standard then suspensions were diluted 1:20 with Middlebrook 7H9 broth containing 10% OADC. Next, 100 μL of each suspension was automatically distributed into a 96-well drug-containing plate using a Sensititre AIM^®^ system (Thermo Fisher Scientific, West Sussex, United Kingdom). Plates were sealed with plastic film then were incubated at 37°C for 10 days. Growth of mycobacteria was monitored using the Sensititre Vizion System (Thermo Fisher Scientific). If insufficient growth was observed in control wells, plates were rechecked after an additional 4 or 11 days of incubation. The minimum inhibitory concentration (MIC) was interpreted using SWIN^®^ software (Thermo Fisher Scientific) and was defined as the lowest concentration inhibiting visual growth of mycobacteria. The reference H37Rv strain was tested in each plate as a quality control measure. LZD concentrations ranged from 0.12 to 8 mg/L as previously described, with a result scored as LZD-resistant for MTB cultures with MIC values greater than 1.0 mg/L (Wasserman et al., 2019). All experiments involving MTB were performed in a biosafety laboratory by authorized and trained researchers. Prior patient exposure to LZD was based on treatment history with LZD-containing regimens. Initial resistance was defined as resistance detected in isolates from patients who had never before received LZD-containing regimens, while acquired resistance was defined as resistance detected in isolates from patients without prior LZD therapy.

### DNA Sequencing

Genomic DNA was extracted from freshly cultured bacteria using a MasterPure^TM^ Complete DNA and RNA Purification Kit (Lucigen Corp., Wisconsin, United States) following the manufacturers’ instructions. A 1:100 dilution of genomic DNA was used as template for performing PCR amplification. The PCR mixture contained 25 μL of 2 × PCR Mixture, 2 μL of DNA template, and 0.2 μM of each primer. PCR thermal cycling was conducted as follows: 5 min at 94°C, followed by 35 cycles each of 1 min at 94°C, 1 min at 58°C, 1 min at 72°C, and one final step of 10 min at 72°C. PCR primers of *rrl*, *rplC*, and *rplD* used herein were synthesized as previously reported (Sharma et al., 2017). After amplification, 40 μL of PCR product for each isolate was sent to the Tsingke Company for DNA purification and sequencing service. DNA sequences were aligned with homologous sequences of reference strain *M. tuberculosis* H37Rv using a multiple sequence alignment tool^2^.

### Statistical Analysis

Data were analyzed following Clinical and Laboratory Standards Institute guidelines. The epidemiological cutoff value (ECV) was defined as the value that encompassed at least 95% of isolates in the wild-type distribution. The significance of intergroup differences (expressed as proportions) was tested using the chi square test. All statistical analyses were conducted using SPSS version 20.0 (IBM, Armonk, New York, United States). A two-sided *P-*value of < 0.05 was considered statistically significant.

## Results

### Distributions of Linezolid MICs for Clinical Isolates

**FIGURE 1:**
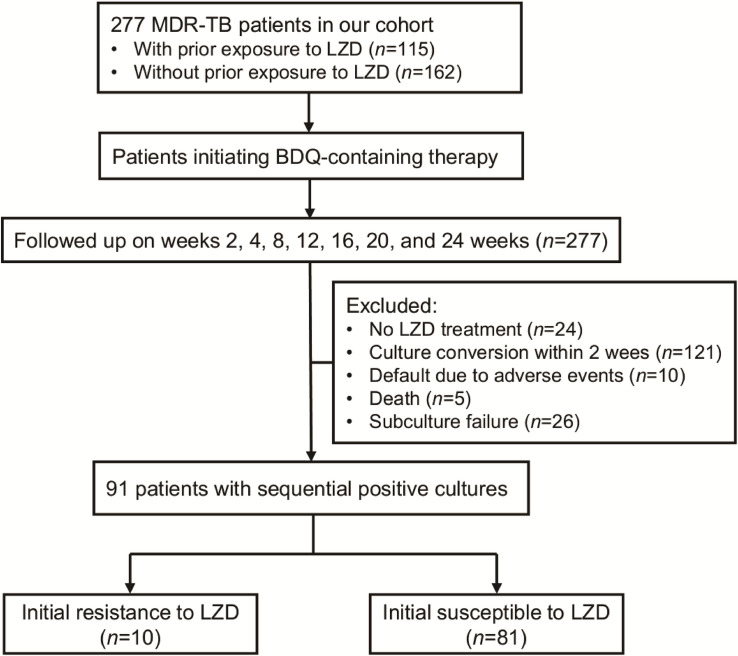
Participant enrollment in this study. A total of 277 MDR-TB patients were included in our cohort. After initiating BDQ-containing therapy, serial cultures of MTB isolates were obtained during follow-up.

**FIGURE 2:**
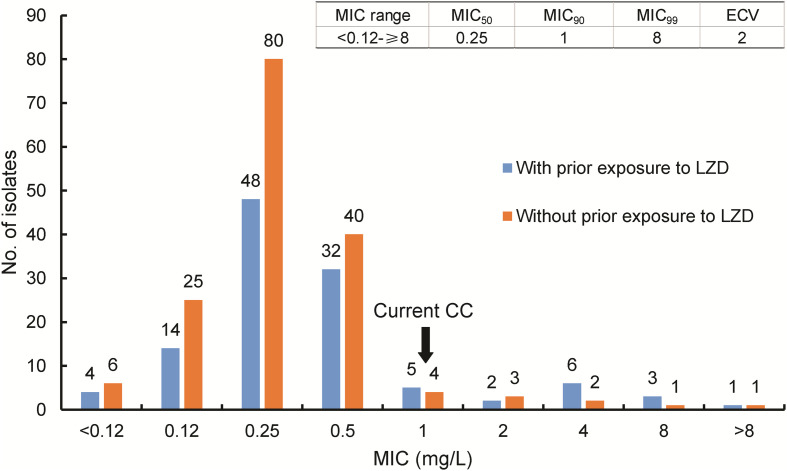
MIC distribution of MDR-TB isolates with varying MIC values stratified by prior LZD exposure history. MIC, minimum inhibitory concentration; ECV, epidemiological cutoff value; CC, critical concentration. The blue column represents results from MTB isolates with prior exposure to LZD; the orange column represents results from MTB isolates without prior exposure to LZD.

A total of 277 patients infected with MDR-TB were included in our cohort. Of these patients, 115 (41.5%, 115/277) had medical histories indicating prior exposures to LZD (Figure 1). MIC distributions are displayed as a histogram in Figure 2 and were stratified based on prior LZD exposure. Considering all 277 clinical isolates, the overall LZD MIC range was 0.12–8 mg/L, with similarly broad frequency ranges observed regardless of prior LZD exposure. The LZD MIC range had an enclosed normal distribution between 0.12 and 8 mg/L for isolates without LZD exposure, while a bimodal distribution was observed for isolates from patients with prior LZD exposure (0.12–8 mg/L). The MIC_90_ of isolates from patients with prior LZD exposure was 2.0 mg/L, a greater value than that obtained for isolates from patients without prior LZD exposure (0.5 mg/L). Based on selection of 1.0 mg/L as the cut-off value, 19 (6.9%, 19/277) isolates resistant to LZD were detected in our MDR-TB cohort that consisted of 12 (63.2%, 12/19) and 7 (36.8%, 7/19) isolates from prior exposed and non-exposed patient groups, respectively. Statistical analysis revealed that the proportion of LZD-resistant isolates in the previously LZD-exposed group significantly exceeded that of the previously non-exposed patient group (*P* = 0.047).

### Mutations Associated With Phenotypic Resistance to Linezolid

Sequencing of *rrl*, *rplC*, and *rplD* was conducted for 19 clinical LZD-resistant isolates. Mutations and corresponding MICs are summarized in Table 1. Genetic mutations were observed in 10 (52.6, 10/19) isolates. A Cys154Arg (36.8%, 7/19) substitution within ribosomal protein L3 was the most prevalent mutation detected in LZD-resistant isolates with LZD MICs of 4–8 mg/L. Meanwhile, the G2270T substitution was the most frequently detected mutation in *rrl* (10.5%, 2/19), followed in prevalence by the substitution of G2270C (5.3%, 1/19). Notably, no isolates harbored dual mutations associated with LZD resistance.

**TABLE 1:** Mutations conferring linezolid resistance in the MTB isolates with initial resistance.

Patient ID	MIC (mg/L)	23S rRNA (*rrl*)*^*a*^*	*rplC*	L3 protein	Previously exposed to LZD	Treatment regimen
						
010050	4	G2270C	WT		No	Bdq/Lzd/Cfz/Cs/Pto/PAS
020003	2	WT	WT		No	Bdq/Lzd/Cfz/Cs/Am/Pto/PAS
030002	8	G2270T	WT		Yes	Bdq/Cs/Cfz/Am/Pto
040012	4	WT	T460C	Cys154Arg	No	Mfx/Bdq/Lzd/Cs/Am/E
040015	4	WT	WT		No	Mfx/Lzd/Cm/Cs/Cfz/Bdq/Z
040021	2	WT	WT		Yes	Mfx/Bdq/Lzd/Cs/Cfz/Cm/PAS
040024	2	WT	WT		No	Mfx/Bdq/Lzd/Cs/Cfz/Am/Z
040037	>8	WT	T460C	Cys154Arg	Yes	Mfx/Bdq/Lzd/Am/Pto
040055	8	WT	T460C	Cys154Arg	Yes	Bdq/Lzd/Cfz/Am/Pto/PAS
050003	4	WT			No	Bdq/Lzd/Cs/Am/PAS
050005	>8	WT	T460C	Cys154Arg	No	Bdq/Lzd/Cs/Am/Pto/PAS
070007	4	WT	WT		Yes	Bdq/Lzd/Cs/Cfz/Cm/Pto
080001	2	WT	WT		Yes	Mfx/Bdq/Lzd/Cfz/Am/Cs/Pto
080007	8	WT	T460C	Cys154Arg	Yes	Mfx/Bdq/Lzd/Cfz/Cm/Cs/Pto
080013	4	G2270T	WT		Yes	Mfx/Bdq/Lzd/Cs/Cm/Pto/Clr
080033	2	WT	WT		Yes	Mfx/Bdq/Lzd/Cs/Cm/PAS
080038	4	WT	T460C	Cys154Arg	Yes	Mfx/Bdq/Lzd/Cs/Cfz/PAS
100019	4	WT	WT		No	Bdq/Lzd/Cfz/Am/Pto/PAS
110002	8	WT	T460C	Cys154Arg	Yes	Mfx/Bdq/Lzd/Cfz/PAS

*WT, wild-type; Bdq, Bedaquiline; Lzd, Linezolid; Mfx, Moxifloxacin; Cfz, Clofazimine; Cs, Cycloserine; Pto, Protionamide; PAS, p-aminosalicylic acid; Am, Amikacin; Cm, Capreomycin; Z, Pyrazinamide; E, Ethambutol; Clr, Claricid.*

### Variation in LZD MIC From the Start of LZD-Containing Therapy

**FIGURE 3:**
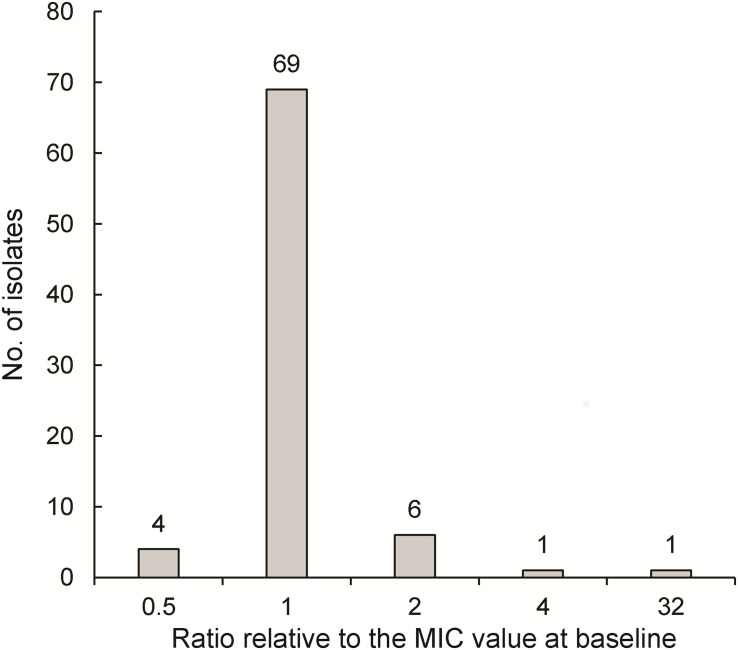
MIC change after LZD exposure (*N* = 81). The horizontal axis represents the ratio of MIC value of the most recently tested isolate prior to culture conversation relative to the MIC value at baseline.

Of 277 patients analyzed for variations in LZD MICs after initiation of LZD-containing regimens, 186 were excluded for various reasons, including prior history without LZD exposure (*n* = 24), culture conversion within 2 weeks (*n* = 121), default exclusion due to adverse events (*n* = 10), death (*n* = 5), and subculture failure (*n* = 26), while another ten cases were excluded due to initial LZD resistance of isolates collected at enrollment. Ultimately, 81 patients with sequential positive cultures from sputa were included for further analysis (Figure 1) by accessing variations in their LZD MICs from the start of LZD-containing therapy throughout the treatment course. As shown in Figure 3, the majority of patients (85.2%, 69/81) retained original LZD MIC values before sputum culture conversion. By contrast, 10 patients treated with BDQ-LZD had a twofold variation of MIC value relative to MIC at baseline, while cultures from another two patients (2.5%) displayed significant increases (≥4-fold) in MICs and became resistant to LZD.

### Patients With Acquired LZD Resistance During Treatment

Detailed demographic and clinical characteristics of the two patients with acquired LZD resistance are listed in Table 2. Although both patients had previously received anti-TB treatment, only one had a history of prior exposure to LZD. Subsequently, LZD resistance for isolates from both patients was detected at 8 and 16 weeks after initiation of LZD treatment. Of note, isolates from Patient 08009 collected at weeks 16, 20, and 24 of treatment showed a consistently elevated LZD MIC value of 4 mg/L, with co-resistance to LZD and BDQ leading to continuous positive culture during therapy. For Patient 040057, despite emergence of LZD resistance, that patient’s isolates remained susceptible to BDQ, with MICs of 0.06 mg/L observed consistently; by the end of the 24-week treatment course, he achieved culture conversion. Sanger sequencing of isolates obtained from these two patients revealed that mutation of *rplC* (T460C base substitution) predominated as the most prevalent acquired LZD resistance mechanism observed for MDR-TB isolates in our patient cohort.

**TABLE 2:** Patients with elevated MICs after exposure to linezolid.

Patient ID	Sex	Age (years)	Patient category	Drug susceptibility	MIC (mg/L)		Mutation in genes associated with LZD resistance			Sputum culture conversion	Previously exposed to LZD	Treatment regimen	Clinical outcome
										
					Baseline	Last^b^	*rrl*	*rplC*	*rplD*				
040057	M^a^	48	Previously treated	Pre-XDR	0.5	2	WT	T460C	WT	12 weeks	No	Bdq/Lzd/Cfz/Cs/Pto/Am	Culture conversion
080009	F	52	Previously treated	XDR	0.12	4	WT	T460C	WT	NA	Yes	Mfx/Bdq/Lzd/Cs/PAS	Culture non-conversion

*^*a*^M, male; F, female; MDR, multidrug-resistance; XDR, extensive drug-resistance; MIC, minimum inhibitory concentration; WT, wild-type.^*b*^Represents the last isolate prior to culture conversation. The last isolates of 040057 and 080009 are obtained 8 and 24 weeks since treatment, respectively.*

## Discussion

The emergence of LZD resistance seriously reduces the efficacy of this agent for use against MTB infections. Based on results for a nationwide MDR-TB cohort, our data found evidence for LZD resistance in 6.9% of MDR-TB patients in China. These results are consistent with previously published data from Beijing demonstrating LZD resistance in 5.4% of MDR-TB patients (Pang et al., 2017). In a recent study from South Africa, 13 (33.3%) of 39 patients with LZD-based treatment failure had phenotypic or genotypic signs of LZD resistance (Wasserman et al., 2019), a rate significantly greater than that found for our cohort. The inclusion of only patients experiencing treatment failure in that study may explain contradictions between those results and results of this study. Of note, here approximately one in ten patients with prior LZD exposure developed resistance to this agent. However, a series of previous studies had demonstrated that LZD resistance was rarely encountered in clinical settings (Richter et al., 2007; Lopez et al., 2019). On the one hand, the identification of LZD-resistant MTB isolates requires implementation of *in vitro* phenotypical susceptibility tests conducted by reliable DSR facilities that are lacking in many facilities that treat patients at high risk for drug-resistance, especially in regions with high TB prevalence (Xu et al., 2018). On the other hand, there is no doubt that the Beijing genotype is the predominant MTB sublineage circulating in China (Pang et al., 2012). Acquisitions of mutations in *mutT4* and *mutT2*, two loci involved in the mismatch repair system, have been identified in certain sublineages of Beijing isolates, thereby leading to higher mutation rates under specific conditions (Ebrahimi-Rad et al., 2003). Consistent with this idea, Zhang et al. (2014) found that Beijing strains were more likely to harbor LZD resistance. Considering that LZD has been endorsed as a priority drug for use in MDR-TB patient treatment by national TB treatment programs, increased vigilance and active surveillance during therapy are urgently needed to guide the formulation of protocols for administering LZD.

Another interesting finding of our study was that 2.5% of LZD naïve patients exhibited LZD resistance, with occurrences of initial resistance reflecting occurrences of past transmission events involving drug-resistant strains. Notably, in view of the fact that LZD has only been used for the clinical treatment of TB for a relative short period of time, observations of initial resistance may be interpreted as an epidemiological indicator associated with recent MDR-TB transmission events. Evidence for this speculation can be found in a regional molecular epidemiological study that concluded that recent transmission of increasingly drug-resistant MDR-TB strains have largely contributed to emergence of the current MDR tuberculosis epidemic in Shanghai (Yang et al., 2017). Taken together, early diagnosis and treatment initiation are essential for reducing future MDR-TB transmission in China.

Combination therapy is clearly indispensable for preventing emergence of drug-resistant organisms (Goldberg et al., 2012). Interestingly, our primary data revealed that low rate of acquired LZD resistance was noted in MDR-TB treated with BDQ-LZD combination with several possible explanations for this noteworthy finding identified here. First, molecular mechanisms responsible for MTB development of resistance to LZD and BDQ during therapy do not overlap (Hameed et al., 2018). Therefore, BDQ in the combination therapy should theoretically remain active against mutants with resistance to LZD and thus should prevent accumulations of mutations conferring LZD resistance. Second, a retrospective clinical study demonstrated that the earliest detected occurrence of LZD resistance in MDR-TB patients occurred 7 months after initiation of LZD treatment, with a median duration before occurrence of LDZ resistance of 22 months (Wasserman et al., 2019). In our cohort, in MDR-TB patients receiving BDQ-containing regimens, culture conversion that was noted in 85% of patients at 20 weeks was attributed to the potent bactericidal activity of BDQ. Therefore, the reduction of time to sputum culture conversion further prevented the emergence of LZD-resistant organisms. Although the mechanism responsible is uncertain, our findings support new WHO treatment recommendations whereby the BDQ-linezolid combination would be prioritized as an MDR-tuberculosis regimen. Meanwhile, two or more additional drugs, such as fluoroquinolones and clofazimine, should be added to this combination to prevent the emergence of drug resistance and clinical failures.

Overall, mutations in *rplC* were the main underlying mechanism for LZD resistance in the majority of our cases; our result aligns with results of previous reports that have repeatedly demonstrated the predominance of this mechanism (Hillemann et al., 2008). Taken together, these results collectively suggest that tubercle bacilli are more likely to harbor *rplC* mutations after exposure to LZD. In an *in vitro* selection study by Beckert et al., the *rplC* T460C substitution was identified as the only polymorphism detected in LZD-resistant clones (Beckert et al., 2012). Similarly, the two isolates with acquired LZD resistance in our cohort also emerged via accumulation of this dominant mutation. Meanwhile, previous results have shown that there is strong selection for drug resistance-conferring mutations associated with low fitness cost (Gagneux, 2009). In view of the universal role of *23S rRNA* as a key participant in protein synthesis, we speculate that *rplC* mutants may incur lower biological fitness costs than would *23S rRNA* mutations, ultimately facilitating survival of LZD-resistant bacteria and their subsequent transmission within the community. Nonetheless, more experimental data are required to verify our hypothesis on fitness costs of LZD resistance.

In addition to genetic mutations within target genes, LZD resistance could also result from cellular changes that negatively influence cell permeability toward antimicrobial agents, including resistance mechanisms involving efflux pumps and changes in cell wall thickness (Sharma et al., 2016; Sharma and Bisht, 2017). In a previous report by Escribano et al. (2007), addition of an efflux pump inhibitor led to significantly decreased MTB susceptibility to LZD. Therefore, we speculate that an active expulsion mechanism may serve as a non-ribosomal resistance mechanism in MTB strains with moderately elevated MICs, as demonstrated in this study. Additionally, an electron microscopic examination of the ultrastructure of the mycobacterial cell wall revealed that cell wall thickness in MTB isolates was positively correlated with extensive drug resistance (Velayati et al., 2009). Thus, low permeability of the mycobacterial cell wall may be another factor conferring LZD resistance, especially in view of the fact that all patients in this study were MDR- and XDR-TB patients. Consequently, more extensive studies should be done in order to elucidate molecular mechanisms conferring LZD resistance among MTB isolates obtained in this study.

Limitations of our study should be mentioned. First, the relatively small number of patients who provided detailed follow-up information weakened the significance of our study. Second, we acknowledge that this study design did not utilize the optimal cohort to conduct a comparative study for observing whether the addition of BDQ would influence the emergence of LZD resistance. However, preferred treatment regimens for MDR-TB patients consist of Group A drugs, including BDQ and LZD. Thus, a self-controlled study may address this issue by allowing testing of our hypothesis. Third, a sizeable proportion of LZD-resistant isolates harbored no mutations within *rrl* and *rplC*, highlighting the need for whole genome sequencing to investigate alternative mechanisms conferring LZD resistance to those isolates. Fourth, our data suggest that BDQ-LZD may be an effective drug combination for preventing emergence of LZD resistance during treatment of MTB infections. Nevertheless, effects of treating MDR-TB patients with this combination treatment on acquired BDQ resistance should also be investigated.

## Conclusion

In conclusion, our data demonstrate that LZD resistance occurred in 6.9% of MDR-TB patients in an MDR-TB cohort in China. Low rate of acquired LZD resistance was noted in MDR-TB treated with BDQ-LZD combination. Moreover, mutations in *rplC* were identified as the underlying mechanism for LZD resistance in the majority of our cases. Further studies are warranted to assess whether use of BDQ-LZD combination treatment would have an analogous effect to the BDQ effect observed here on LZD resistance, whereby LZD treatment would instead affect acquired BDQ resistance in MDR-TB patients.

## Data Availability Statement

The datasets presented in this study can be found in online repositories. The names of the repository/repositories and accession number(s) can be found below: NCBI BioSample, accession nos: SAMN18449982, SAMN18449983, and SAMN18449984.

## Ethics Statement

This study was approved by the Ethics Committee of Beijing Chest Hospital, Capital Medical University. The patients/participants provided their written informed consent to participate in this study.

## Author Contributions

LL and YP designed of the work. JD, JG, and YP wrote the manuscript. YY, QL, GB, LW, YW, ZX, and FH detected strain. JG, MG, and YL collected the data. JD and WS analyzed the data. All authors approved the final version to be submitted for consideration for publication.

## Conflict of Interest

The authors declare that the research was conducted in the absence of any commercial or financial relationships that could be construed as a potential conflict of interest.

## Footnotes

1 https://ndip.chinaiatb.org/static/index.html#/login

2 http://www.ncbi.nlm.nih.gov/BLAST
